# Locked intramedullary nailing of open fractures in resource-poor settings: a prospective observational study of challenges and functional outcomes in 101 fractures at Ogbomoso, Nigeria

**DOI:** 10.1186/s12891-023-06271-7

**Published:** 2023-03-07

**Authors:** Stephen Adesope Adesina, Isaac Olusayo Amole, James Idowu Owolabi, Oluwafemi Oyewole Oyewusi, Imri Goodness Adefokun, Samuel Uwale Eyesan

**Affiliations:** 1 Bowen University Teaching Hospital, P. O. Box 15, Ogbomoso, Oyo State Nigeria; 2 Bowen University, P.M.B 284, Iwo, Osun State Nigeria

**Keywords:** Open fracture, Infection, Locked intramedullary nail, Long bone fracture, Low- and middle-income countries

## Abstract

**Background:**

Trauma is now one of the fastest growing epidemics globally but low and middle-income countries (LMICs) are more severely affected in terms of cost, disability and death. The high-energy trauma of road traffic accidents and violence often produces open fractures which can be difficult to manage in resource-poor settings. Adequate stabilization, such as provided by locked nails, has been found to ensure better outcome for open fractures. There is dearth of published studies on the use of locked intramedullary nail in the treatment of open fractures in Nigeria.

**Methods:**

This is a prospective observational study of all the 101 open fractures of the humerus, femur and tibia treated over a period of 92 months with Surgical Implant Generation Network (SIGN) nail. Fracture severity was classified according to the modified Gustilo-Anderson system. The intervals between fracture and antibiotics administration, débridement and definitive fixation, as well as surgery duration and method of fracture reduction were noted. Outcomes measured at follow-up included infection, ongoing radiographic healing, knee flexion/shoulder abduction beyond ninety degrees (KF/SA > 90^0^), full weight bearing (FWB), painless squatting (PS&S)/shoulder abduction-external rotation (SAER).

**Results:**

Most of the patients fall between ages 20 and 49 years; 75.5% of them were males. There were more Gustilo-Anderson type IIIA fractures than other types but nine type IIIB tibia fractures were also nailed. The overall infection rate was 15%, contributed mostly by the type IIIB fractures. By the 12th post-operative week, at least 79% had ongoing radiographic healing and had achieved all of KF/SA > 90^0^, FWB, and PS&S/SAER.

**Conclusion:**

The SIGN nail’s solid construct reduces the risk of infection and allows earlier use of the limb, making it particularly suitable in LIMCs where socioeconomic functioning often requires an unhindered use of the limbs.

## Background

Trauma is now one of the fastest growing epidemics globally but developing countries are more severely affected in terms of cost, disability and death [1]. In low and middle-income countries (LMICs), road traffic accidents (RTAs) alone are responsible for a quarter of all injuries while some more are caused by incessant violent incidents [2–4]. Injuries sustained in RTAs and violent crisis are mostly musculoskeletal in nature, fracture being chief among them, with an estimated 80% of severe fractures occurring in the developing world [1, 2]. Although fractures can occur in an intact soft tissue envelope, the high-energy trauma of RTAs and violence often results in open fractures with varying degrees of soft tissue and skeletal injury [5, 6].

The soft tissue and skeletal injuries in open fractures can make their management especially challenging even in high-income countries (HICs) with advanced trauma systems, state-of-the-art equipment and modern management principles/techniques. This is due to the exposure of fracture haematoma to microbial contamination and impaired local tissue vascularity with consequent increased risk of infection as well as complications in healing [5, 6]. Thus, management goals in open fractures are prevention of infection, union of the fracture, and restoration of function [6]. To achieve these goals, there is need for early administration of antibiotics, thorough irrigation and débridement of the wound, fracture stabilization and reconstruction of the soft tissue covering of exposed bone [5, 6]. Achieving all of these can be quite difficult in the austere settings of many LMICs, and it has been aptly noted that outcomes are particularly bad for open fractures in these countries [7].

The bad outcomes are due to challenges such as absence of trauma care delivery systems, patients’ late presentation to orthopaedic specialists, out-of-pocket payment for care, inadequate staffing of hospitals, lack of education and training, outdated theatre facility and equipment, lack of modern implants and consumables, intermittent water and power supply, and so on [4, 7, 8]. Of these difficulties, access to modern implants and equipment for stable fixation of the fractures appears to be the hardest barrier to surmount [1]. Modern implants are not readily available and patients must often pay for whichever is found out-of-pocket at a cost beyond their reach [7]. Otherwise, outdated techniques, such as prolonged traction or casts are used to treat open long bone fractures [9].

Yet, the importance of adequate stabilization with appropriate implants in ensuring healing of open fractures has been well-established: apart from protecting the soft tissues from further injury by fracture fragments, it also facilitates the host response to bacteria, improves wound care, affords early joint motion and mobilization of the patient, and thus, ultimately contributing to functional rehabilitation [6]. Operative stabilization methods include intramedullary (IM) nailing, external fixation, and plate-and-screw fixation. While the method of choice ultimately depends on the anatomic location of the fracture and the extent of soft-tissue injury, IM nailing is considered preferable to external fixation for types I, II and IIIA fractures as it does not demand the same high level of patient compliance and it is aesthetically more acceptable than external fixation [6]. As for plate fixation, increased incidence of infection and implant failure have been the discouraging events [10, 11].

Many previously published studies in LMICs on the use of locked IM nail for the treatment of long bone fractures included only very small numbers of open fractures [11–14], while others excluded them altogether [14–17]. Earlier open fracture studies in Nigeria mixed the outcomes of the many stabilization methods (IM nailing, external fixation, K-wires, plate and screw, and casting) and did not specify the type of IM nail used [3, 4, 18]. The aim of this study was to discuss the challenges encountered in the treatment of 101 open fractures (in 94 patients) in our centre with locked IM nail and to present the outcomes in terms of occurrence of infection and functional recovery.

## Methods

The study centre is a teaching hospital in South-western Nigeria which serves the people of Ogbomoso and nearby villages and towns that were home to subsistence farmers, small business owners, civil servants and artisans. The means of transportation were huge numbers of motorcycles, tricycles and increasing number of used/salvage vehicles imported from the Western HICs. Using an observational study design, data were collected prospectively on all the 101 open fractures of the humerus, femur and tibia treated between July 2014 and February 2022 (92 months) with the solid reamed locked intramedullary nail (IM) manufactured and freely donated by Surgical Implant Generation Network (SIGN, Richland, WA, USA). The inclusion criteria were open fractures of the humerus, femur and tibia treated with the SIGN nail between July 2014 and February 2022. The exclusion criteria were open fractures treated by means other than SIGN nailing, and open fractures whose skin wounds had healed before presenting to us. The data included patient and fracture characteristics, treatment details and outcome.

Fracture severity was classified according to the modified Gustilo-Anderson system [5, 6, 19]. *Type I* = puncture wounds ≤ 1 cm, with minimal contamination and muscle damage. *Type II* = lacerations > 1 with moderate soft-tissue injury, adequate bone coverage and minimal comminution. *Type IIIA* = extensive soft-tissue damage from high-velocity injury with severe crushing component, heavily contaminated wounds, severe comminution/segmental fractures but adequate bone coverage. We included gunshot fractures here. We presumed fractures that had been open for ≥ 8 h prior to treatment to be heavily contaminated and classified them as type IIIA. *Type IIIB* = extensive soft-tissue damage, with stripping of the periosteum and exposure of the bone, usually associated with heavy contamination and severe comminution of the bone, and inadequate soft tissue cover. *Type IIIC* = any open fracture with arterial injury requiring repair, regardless of the degree of soft-tissue injury.

The patients were started on broad spectrum antibiotics on arrival in our Emergency Room (ER) and had the fractured lower limb splinted with plastic back-slab or upper limb with sling. Once assessed fit for anaesthesia following initial resuscitation, wound débridement and irrigation were done in the operating room (OR). For patients who presented ≤ 8 hours’ post-injury, immediate primary wound closure was done if there was no soft tissue loss. Otherwise, a delayed closure with muscle flap and split-thickness skin graft was done at the time of definitive fracture fixation. Depending on the severity of their injuries, the patients either had further OR débridement or daily wound dressing changes by nurses on the wards. Definitive fracture fixations were done with the SIGN nail any time from day 0 post-injury, using the surgical procedure described by the manufacturer [20–22]. When possible, closed reduction was done for fractures where primary wound closure had been done; otherwise, open or finger reduction was done [23]. Post-operatively, the patients were continued on intravenous broad spectrum antibiotics for five to seven days and subsequently on oral antibiotics until their wounds healed.

The time length between skin incision and closure (*duration of surgery*) was categorized into: ≤1 h, >1 h but ≤ 2 h, >2 h but ≤ 3 h, and > 3 h. The time length between occurrence of fracture and antibiotics administration (*fracture-to-antibiotics interval*) was categorized into: ≤3 h, > 3 but ≤ 6 h, and > 6 h. The time length between occurrence of fracture and wound débridement (*fracture-to-débridement interval*) was categorized into: ≤6 h, > 6 but ≤ 24 h, and > 24 h. The time length between occurrence of fracture and definitive fracture fixation (*fracture-to-fixation-interval)* was categorized into 0–2, 3–7, and > 7 days. The fractures were further classified into open, closed and finger reduction categories. The patients were grouped according to post-operative length of hospitalization as: died on admission, discharged in post-operative week one, week two or after week two. Pre- and post-operative radiographs were taken. As their conditions permitted, patients were made to ambulate beginning from post-operative day one, and discharged from the hospital starting from post-operative day five.

Post-discharge, they were followed-up with plain radiographs and tests of ability to do knee flexion/shoulder abduction beyond ninety degrees (KF/SA > 90^0^), full weight bearing (FWB), painless squatting and smiling ([PS&S], femur/tibia) or shoulder abduction-external rotation ([SAER], humerus). For all patients, the follow-ups were done at least twice — at six and 12 weeks. If FWB was not achieved or there was no radiological evidence of ongoing healing at the 12th week, a patient was expected to return at the 6th post-operative month. If infection occurs, additional follow-up visits were scheduled based on the healing progress. All the patients were also instructed to return if they had pain or discharge from their operated limb. Infection was defined as presence of purulent discharge from (or near) the incision necessitating sustained antibiotic therapy and surgical debridement and/or implant removal. The study protocols were approved by Bowen University Teaching Hospital Research Ethics Committee. All patients aged ≥ 16 years and the parents (or legal guardians) of those < 16 years gave informed consent to be included in the study. All methods were carried out in accordance with relevant guidelines and regulations.

### Statistical analysis

The data were analysed with SPSS version 23 (IBM Corp, New York, USA). The fracture severity (type) was cross-tabulated with infection and Fisher’s exact test was used to determine if there was any significant association between the two. All *p* values were two-tailed and the level of significance was set at *p* < 0.05.

## Results

**Fig. 1:**
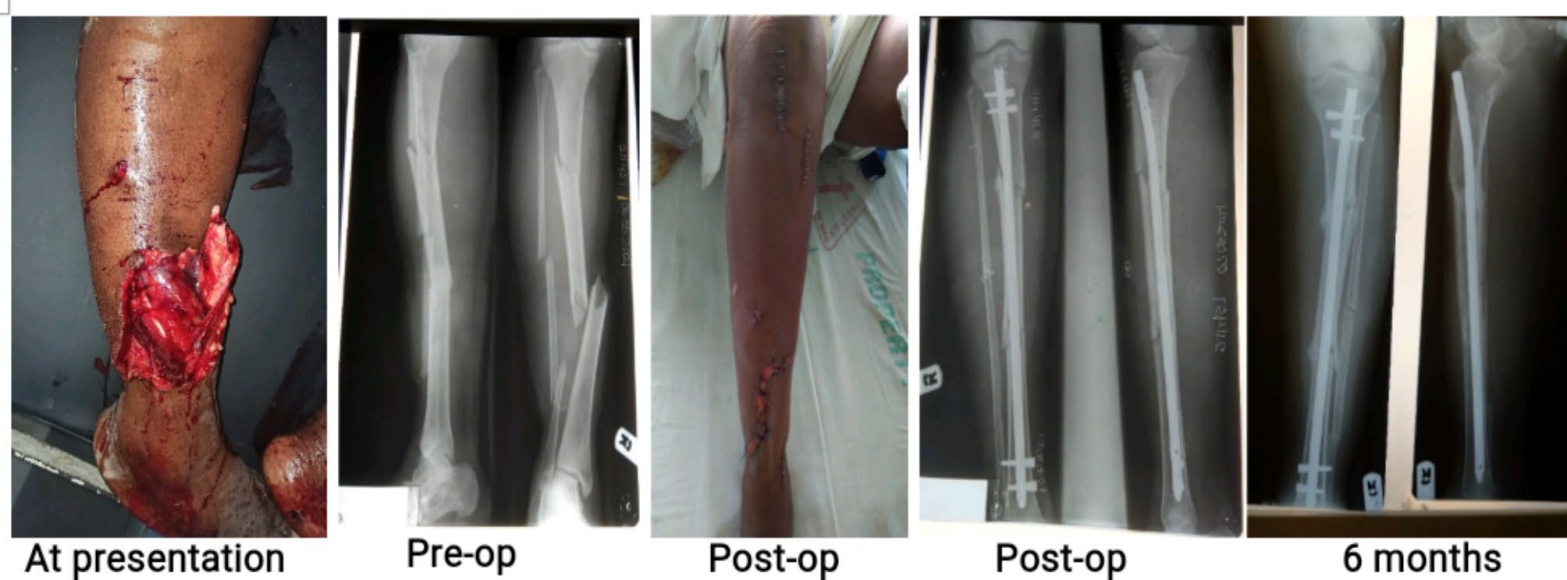
Photographs of a 51-year old woman who sustained type IIIA tibia fracture in pedestrian accident. Wound debridement, immediate primary closure and locked intramedullary nailing were done within 6 h of presentation. The fracture healed without infection

**Fig. 2:**
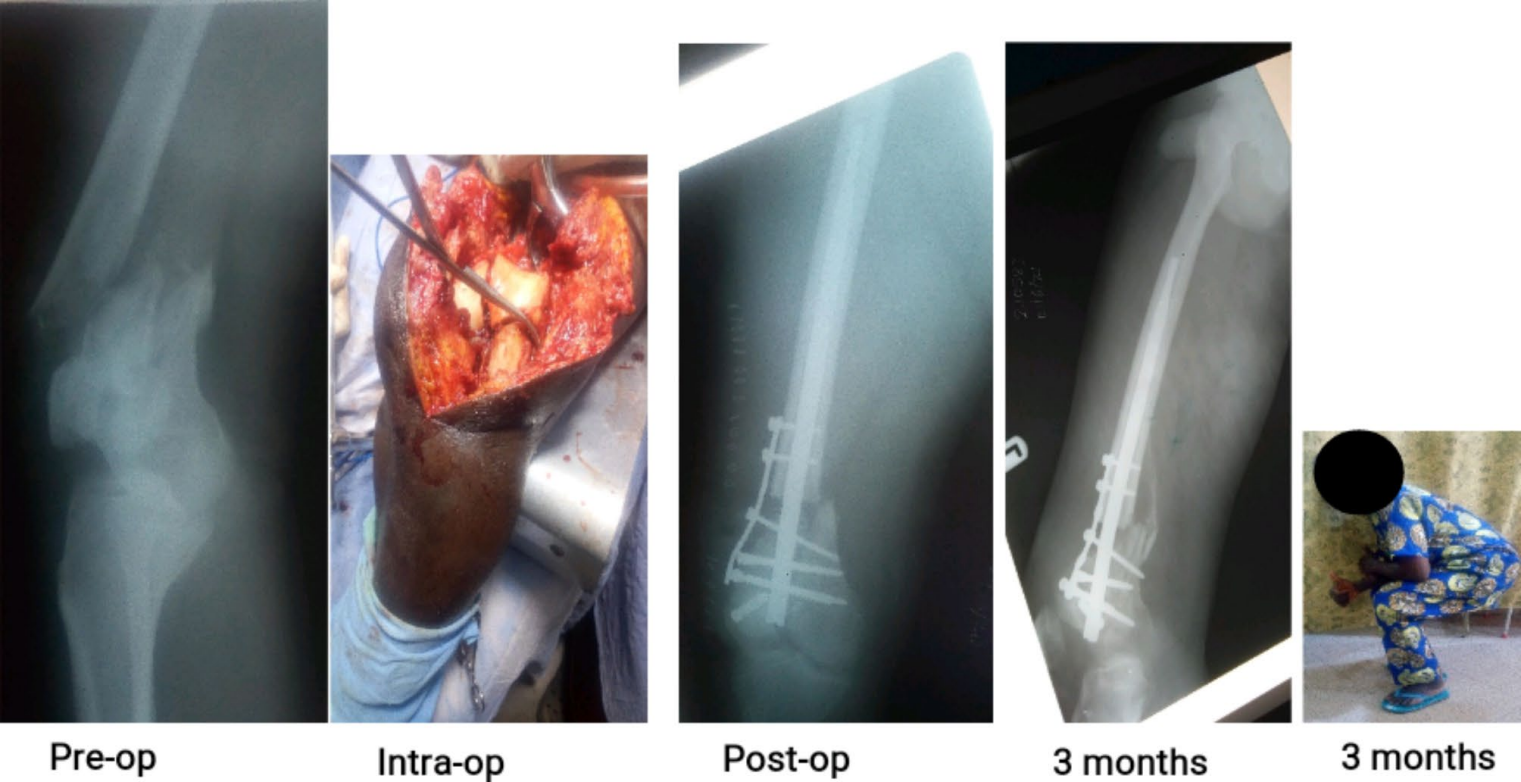
Photographs of a 37-year old man who sustained type IIIA multifragmentary distal articular femur fracture and traumatic brain injury in motorcycle accident. Wound debridement and immediate primary closure were done on the day of injury. Stabilization with a locked intramedullary nail and augmenting side plate was done 9 days post-injury after he recovered from head injury. The fracture healed without infection

**Fig. 3:**
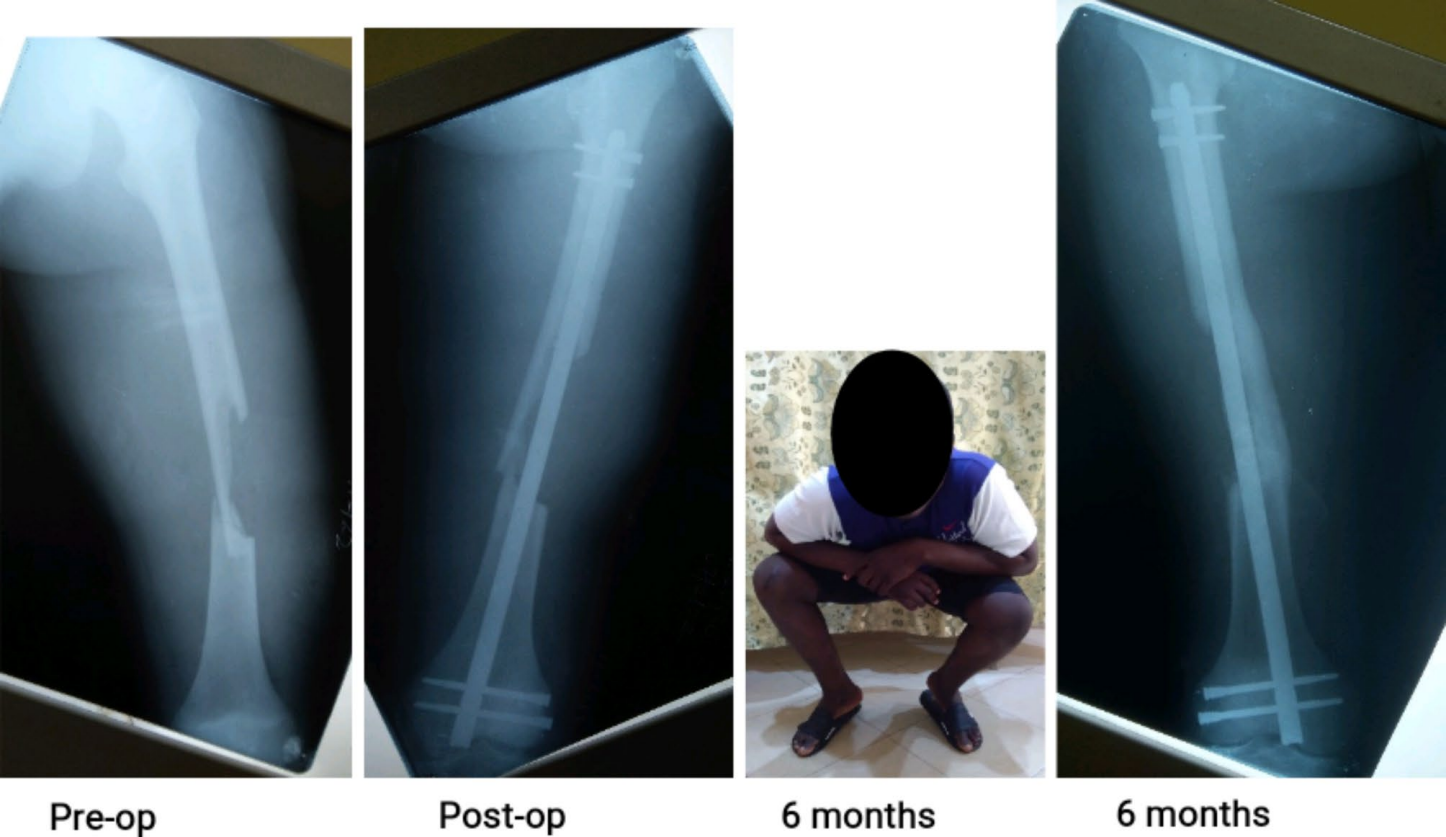
Photographs of a 16-year old boy who sustained type IIIA femur fracture in motorcycle accident. The large butterfly fragment fell out of the wound at the accident scene. Wound debridement and immediate primary closure were done on the day of injury. Locked intramedullary nail was done 3 days post-injury. The fracture healed without infection

Over the study period, 101 open fractures were definitively stabilized with the SIGN IM nail, accounting for 22% of a total of 460 fresh fractures nailed. The 101 were made up of 2 (2.0%) humerus, 40 (39.6%) femur and 59 (58.4%) tibia fractures. Figures 1, 2 and 3 are clinical photographs of some of the patients included in this study.

**Table 1:** Patients’ characteristics and cause of fracture

Variables (N = 94)		n (%)
Age groups (years) *Mean age = 37.68 years* *Age range: 14–76 years*	10–19	7 (7.5)
	20–29	20 (21.3)
	30–39	27 (28.7)
	40–49	24 (25.5)
	50–59	9 (9.6)
	60–69	5 (5.3)
	70–79	2 (2.1)
Gender	Male	71 (75.5)
	Female	23 (24.5)
Occupation	Artisans and farmers	30 (31.9)
	Civil servant and clergy	21 (22.4)
	Commercial drivers/motorcycle riders	16 (17.0)
	Small business owners	16 (17.0)
	Student and dependants	11 (11.7)
Cause of fracture	Gunshot	1 (1.1)
	Fall	1 (1.1)
	Motor vehicle accident	19 (20.2)
	Motorcycle accident	59 (62.8)
	Pedestrian accident	14 (14.9)

Table 1 shows age group 30–39 years was the modal age group although most of the patients fall between ages 20 and 49 years. More than three-quarters (75.5%) of the patients were males. Many (31.9%) were artisans or farmers and motorcycle accident was responsible for more than three-fifths (62.8%) of the fractures.

**Table 2:** Fracture types and treatment details

Variables		Humerus (n = 2) n (%)	Femur (n = 40) n (%)	Tibia (n = 59) n (%)	Total (N = 101) n (%)
Gustilo type	Type I	0 (0.0)	10 (25.0)	20 (33.9)	**30 (29.7)**
	Type II	0 (0.0))	7 (17.5)	13 (22.0)	**20 (19.8)**
	Type IIIA	2 (100.0)	23 (57.5)	17 (28.8)	**42 (41.6)**
	Type IIIB	0 (0.0)	0 (0.0)	9 (15.3)	**9 (8.9)**
	Type IIIC	0 (0.0)	0 (0.0)	0 (0.0)	**0 (0.0)**
Fracture-to-antibiotics interval	≤ 3 h	0 (0.0)	19 (47.5)	32 (54.2)	**51 (50.5)**
	> 3 but ≤ 6 h	1 (50.0)	15 (37.5)	16 (27.1)	**32 (31.7)**
	> 6 h	1 (50.0)	6 (15.0)	11 (18.6)	**18 (17.8)**
Fracture-to-débridement interval	≤ 6 h	1 (50.0)	26 (65.0)	42 (71.2)	**69 (68.3)**
	> 6 but ≤ 24 h	1 (50.0)	14 (35.0)	15 (25.4)	**30 (29.7)**
	> 24 h	0 (0.0)	0 (0.0)	2 (3.4)	**2 (2.0)**
Fracture-to-fixation-interval	0–2 days	0 (0.0))	9 (22.5)	22 (37.3)	**31 (30.7)**
	3–7 days	1 (50.0)	19 (47.5)	28 (47.5)	**48 (47.5)**
	> 7 days	1 (50.0)	12 (30.0)	9 (15.3)	**22 (21.8)**
Fracture reduction method	Open	2 (100.0)	22 (55.0)	22 (37.3)	**46 (45.5)**
	Closed	0 (0.0)	14 (35.0)	37 (62.7)	**51 (50.5)**
	Finger	0 (0.0)	4 (10.0)	0 (0.0)	**4 (4.0)**
Duration of surgery	≤ 1 h	1 (50.0)	13 (32.5)	32 (54.2)	**46 (45.5)**
	> 1 but ≤ 2 h	1 (50.0)	22 (55.0)	22 (37.3)	**45 (44.6)**
	> 2 h	0 (0.0)	4 (10.0)	5 (8.5)	**10 (9.9)**
Length of Post-operative hospital stay	Died	0 (0.0)	0 (0.0)	1 (1.7)	**1 (1.0)**
	Discharged week 1	2 (100.0)	33 (82.5)	48 (81.4)	**83 (82.2)**
	Discharged week 2	0 (0.0)	5 (12.5)	2 (3.4)	**7 (6.9)**
	Discharged after week 2	0 (0.0)	2 (5.0)	8 (13.6)	**10 (9.9)**

The analysis displayed in Table 2 reveals that there were more Gustilo-Anderson type IIIA fractures than any other type. It is noteworthy, however, that nine type IIIB tibia fractures were also nailed. The first dose of antibiotics was administered within the first 3 three hours of injury in one-half (50.5%) of the fractures while more than two-thirds (68.3%) had irrigation and débridement within the first 6 h. The fractures were mostly (47.5%) fixed definitively between day three and seven post-injury and 50.5% of them had closed reduction. Only ten (9.9%) of the surgeries lasted more than 2 h and majority (82.2%) were discharged home in the first post-operative week. One patient died of pulmonary embolism while on admission.

**Table 3:** Treatment outcomes

Variables		Humerus (n = 2) n (%)	Femur (n = 40) n (%)	Tibia (n = 58) ^x^ n (%)	Total (N = 100) ^x^ n (%)
Infection	No	1 (50.0)	35 (87.5)	49 (84.5)	**85 (85.0)**
	Yes	1 (50.0)	5 (12.5)	9 (15.5)	**15 (15.0)**
Ongoing radiographic healing noted at:	6 weeks	1 (50.0)	33 (82.5)	42 (72.4)	**76 (76.0)**
	12 weeks	0 (0.0)	5 (12.5)	16 (27.6)	**21 (21.0)**
	After repeat surgery	0 (0.0)	1 (2.5)	0 (0.0)	**1 (1.0)**
	Not achieved	1 (50.0)	1 (2.5)	0 (0.0)	**2 (2.0)**
Knee flexion/Shoulder abduction > 90 degrees (KF/SA > 90^0^) present at:	6 weeks	1 (50.0)	17 (42.5)	46 (79.3)	**64 (64.0)**
	12 weeks	0 (0.0)	17 (42.5)	8 (13.8)	**25 (25.0)**
	6 months	1 (50.0)	2 (5.0)	3 (5.2)	**6 (6.0)**
	After 6 months	0 (0.0)	3 (7.5)	1 (1.7)	**4 (4.0)**
	Not achieved	0 (0.0)	1 (2.5)	0 (0.0)	**1 (1.0)**
Full weight-bearing (FWB) noted at:	6 weeks	1 (50.0)	14 (35.0)	25 (43.1)	**40 (40.0)**
	12 weeks	0 (0.0)	22 (55.0)	28 (48.3)	**50 (50.0)**
	6 months	0 (0.0)	2 (5.0)	5 (8.6)	**7 (7.0)**
	After 6 months	0 (0.0)	1 (2.5)	0 (0.0)	**1 (1.0)**
	Not achieved	1 (50.0)	1 (2.5)	0 (0.0)	**2 (2.0)**
Able to squat and smile (PS&S/SAER) at:	6 weeks	1 (50.0)	10 (25.0)	22 (37.9)	**33 (33.0)**
	12 weeks	0 (0.0)	22 (55.0)	24 (41.4)	**46 (46.0)**
	6 months	1 (50.0)	4 (10.0)	9 (15.5)	**14 (14.0)**
	After 6 months	0 (0.0)	3 (7.5)	3 (5.2)	**6 (6.0)**
	Not achieved	0 (0.0)	1 (2.5)	0 (0.0)	**1 (1.0)**

^x^ One patient who died on admission was excluded from this analysis

It is noted in Table 3 that the overall infection rate was 15%. By the 12th post-operative week, a total of at least 79% had ongoing healing on plain radiograph and had achieved all of KF/SA > 90^0^, FWB, and PS&S/SAER.

**Table 4:** Cross tabulation of infection with Gustilo-Anderson type

Bone	Gustilo-Anderson type	Infection		p-value ^z^
		No **n (%)**	Yes **n (%)**	
Humerus (n = 2)	Type I	0 (0.0)	0 (0.0)	*-*
	Type II	0 (0.0)	0 (0.0)	
	Type IIIA	1 (50.0)	1 (50.0)	
Femur (n = 40)	Type I	9 (90.0)	1 (10.0)	*1.000*
	Type II	6 (85.7)	1 (14.3)	
	Type IIIA	20 (87.0)	3 (13.0)	
Tibia (n = 58) ^x^	Type I	20 (100.0)	0 (0.0)	0.000*
	Type II	12 (92.3)	1 (7.7)	
	Type IIIA	15 (93.8)	1 (6.3)	
	Type IIIB	2 (22.2)	7 (77.8)	
All fractures (N = 100) ^x^	**Type I**	**29 (96.7)**	**1 (3.3)**	***0.000*** *
	**Type II**	**18 (90.0)**	**2 (10.0)**	
	**Type IIIA**	**36 (87.8)**	**5 (12.2)**	
	**Type IIIB**	**2 (22.2)**	**7 (77.8)**	

^x^ One patient who died on admission was excluded from this analysis

^z^ Fisher’s exact test

* Statistically significant values

In Table 4, it is observed that fracture severity had a statistically significant association with occurrence of infection, and that the type IIIB tibia fractures were responsible for this significance.

## Discussion

The demographic distribution of the patients in this study is particularly threatening as the victims were mostly male (75.5%) in the most economically productive age groups 20 to 49 years, purporting that these injuries will further impoverish the population (Table 1). Of the 460 fresh fractures treated, 101 (22%) were open fractures (humerus − 2.0%; femur − 9.6%; tibia − 58.4%). This finding is in keeping with reports of earlier studies that open fractures are more common in the tibia because of the subcutaneous situation of its anteromedial surface [24]. The relatively high proportion of open fractures among our patients, one-half (50.5%) of which were the severe forms (type III), was not surprising since majority of the fractures were caused by high-energy trauma [6], motorcycle accident being chief among them. In earlier studies that combined both closed and open fractures [23, 25], motorcycle accidents accounted for lesser percentages of the fractures than the much higher proportion (62.8%) they contributed in the present study. This suggests that motorcycle accidents cause more severe injuries than other forms of accidents in our environment.

When the foregoing is placed against the backdrop of the care challenges in the austere setting in which these fractures occur, the grave situation in many LMICs like ours become more apparent. In our setting for example, the number of patients who present to the hospital is small compared to the actual number of victims because of poverty-induced pervasive patronage of traditional bone setters where many end up with poverty-propagating abnormal fracture unions [26]. Many of those who present arrive the hospital later than what is obtainable in HICs owing to a grossly inadequate Emergency Medical Services. In this study, about one-half (49.5%) of the fractures arrived the hospital latter than three hours’ post-injury, using the time of antibiotics administration to estimate the time length between fracture and hospital arrival.

Moreover, the resources – both human and material – that were available in the hospitals for the care of these fractures were insufficient. At the time of writing this manuscript, only one orthopaedic surgeon in his late sixties was available in our hospital; others had either emigrated to the western HICs or left for ‘greener pastures’ within Nigeria. A government-owned tertiary hospital in the city has four orthopaedic surgeons but does not have free locked IM implants. Many initial wound débridements and closures were done by junior non-orthopaedic residents. Although he was meticulously trained by the elderly orthopaedic surgeon mentioned earlier and indirectly by SIGN Fracture Care International, the lead author, a family physician, was the lead ‘surgeon’ in virtually all the definitive locked nailing, assisted mostly by junior non-orthopaedic residents or medical officers. In an ideal setting, specialist surgeons handle the most serious open injuries, but the fact that these injuries present to any doctor providing emergency care in LMICs suggests a universal understanding of their management is essential [5].

The overall infection rate in this study was 15% (Table 3) and the cross-tabulation in Table 4 indicates that Gustilo types I, II, IIIA and IIIB respectively had 3.3%, 10.0%, 12.2% and 77.8% infection rates. This association was statistically significant (p < 0.001) but a closer look at Table 4 shows that the very high infection rate among type IIIB fractures (seven out of nine) was responsible for the significance. (The infection cleared with antibiotics and nail removal following fracture healing). For types I to IIIA, the infection rates were comparable to earlier documented rates of 0–2%, 2–10% and 10–50% for type I, II and III respectively [6, 27]. In an evaluation of 1061 open tibia fractures treated with the SIGN nail at many centres in LMICs, Whiting et al. [24] reported infection rates of 5.1%, 12.6% and 12.5% respectively for types I, II and IIIA. We opine that our approach to treatment, detailed in Table 2 contributed to the relatively lower infection rate we recorded. Early antibiotic administration [27, 28], prompt thorough irrigation/débridement with immediate primary wound closure [6, 24, 29, 30], early definitive fracture stabilization [5, 6, 24], and closed (or limited open) reduction [1, 23], are all known to reduce infection rate.

As for the high infection rate in type IIIB fractures, we identified a number of factors that could have been responsible. These included the more severe soft-tissue injury, delayed wound closure and delayed nailing (the only two uninfected cases were nailed within the first week). It has been previously established that the risk of infection increases with increasing severity of soft injury as defined by Gustilo-Anderson type [6, 24, 27]. Further, our lack of reliably efficient sterile dressings (such as antibiotic bead pouch and vacuum assisted closure) for wounds planned for delayed closure made immediate primary wound closure the plausible alternative, which incidentally was observed to reduce infection rate. Indeed, findings of many recent studies supported immediate primary wound closure [24,29.30], but we could not do this for the type IIIB fractures. Similarly, increased time from injury to definitive bony stabilization was found to be associated with a significantly higher infection rate by Whiting et al. [24].

As noted by a previous study [6], an external fixator might have been particularly useful for the types IIIB fractures. Its use however, is not completely innocuous as it may be complicated by pin tract infections, fracture malalignment with resultant mal-/non-union, and there is often the need for transition to another form of fixation which is known to increase the risk of infection [31, 32]. Implementing the protocols (e.g. use of half-pins inserted after predrilling to avoid thermal necrosis of bone, meticulous care of the pin tract and a careful selection of compliant patients) to avoid these complications in our grim setting of low education and poverty is grueling or not feasible. Worse still, apart from the very few donated old pin-to-bar unilateral external fixators, the SIGN nail was the only other implant available free of charge. In our setting, people accept external fixation reluctantly only as a last resort; it is neither socially acceptable nor economically reasonable owing to its need for prolonged hospital stay or multiple surgeries. Since they earned wages predominantly by physical labour (see occupation in Table 1), many of our patients preferred an infected but healed fracture with the early functional restoration of their limb afforded by nailing to a sterile non-union achievable by protracted treatment with an external fixator.

Apart from infection, other outcome measures in this study included the time we noted evidence of ongoing healing on plain radiograph, and the time KF/SA > 90^0^, FWB, and PS&S/SAER were achieved. Table 3 shows that a total of at least 79% had achieved all of these by the 12th post-operative week. Intramedullary fixation of open fractures offers many benefits such as earlier mobilization and avoidance of protracted bed rest with its attending complications including orthostatic pneumonia, pressure sores and venous thromboembolism [33]. It is also associated with less chance of infection and shorter healing time than external fixation [34]. Consequently, it has become the standard definitive treatment method in developed countries, replacing the use of casting and external fixation [35, 36]. The infection rates for types I to IIIA fractures in our study compared favorably with those reported from HICs while the rate for the severe open fractures (type IIIB) was far higher [6, 19, 27, 34, 35]. This highlights the inadequacy of our resources to handle such fractures and the need to improve upon our management protocol for severe soft-tissue injury associated with such fractures.

## Conclusion

In conclusion, the SIGN nail’s solid construct presents less surface area for microbial adherence and also makes it stronger than a cannulated nail of the same diameter [24], thereby reducing the risk of infection and allowing for earlier functional restoration of the limb. These advantages are particularly desirable in LMICs where socioeconomic functioning often requires an unhindered use of the limbs for gainful employment and poverty alleviation [9]. It is deducible from our study that locked IM nailing of open fractures in resource-poor settings do not necessarily produce higher infection rates than those of advance economies, especially if antibiotics are administered early and definitive fracture stabilization is not delayed beyond the first week of injury.

## Data Availability

The datasets used and/or analysed during the current study are available from the corresponding author on reasonable request.

## List of abbreviations

LMICs: low and middle-income countries
RTAs: Road Traffic Accidents
HICs: high-income countries
IM: Intramedullary
SIGN: Surgical Implant Generation Network
ER: Emergency Room
OR: Operating Room
KF/SA > 90^0^: knee flexion/shoulder abduction beyond ninety degrees
FWB: full weight bearing
PS&S: painless squatting and smiling
SAER: shoulder abduction-external rotation
